# Children in Workhouses

**Published:** 1920-09-25

**Authors:** 


### Children in Workhouses.

An interesting feature of the first annual report of the Ministry of Health (a summary of which has already appeared in The Hospital) has been scarcely touched upon by any of the daily newspapers. It is that which relates to children in workhouses, and it raises an important question of accommodation that vitally affects the modern principle of dealing with such children. The Ministry records, in the first place, that the removal of children over three years of age from workhouses was made obligatory by the Poor-Law Institutions Order of 1913, but there were a number of unions which had been unable to make the necessary readjustments by the time of the outbreak of war. The conditions of war-time, and especially the necessity of limiting to the utmost any capital expenditure, made it impossible to proceed with the matter at that time, and temporary dispensations were allowed from the requirements of the Order in the case of some 150 unions.

Since last September all unions in which the Order was not being observed have been pressed to take immediate action. There are at the present time special difficulties in the way of the provision of separate children's homes, especially in view of the shortage of houses, and it is generally impossible for Guardians to erect new houses for this purpose. In some cases Guardians have had to purchase or give up premises of which they were already tenants, and in others they are unable to secure possession, now that the houses could be brought into use as children's homes. In view of these difficulties Guardians have been urged to adopt other methods, especially the boarding-out system and the use of spare accommodation in certified schools and homes belonging to neighbouring unions.

